# Early percutaneous thrombolysis for AVF thrombosis: symptom duration as a predictor of endovascular salvage

**DOI:** 10.1186/s42155-025-00587-2

**Published:** 2025-08-19

**Authors:** Ismail Taskent, Guler Gulsen Ersoy

**Affiliations:** 1 Department of Radiology, Faculty of Medicine, Kastamonu University, Kastamonu, Türkiye; 2 Department of Cardiovascular Surgery, Faculty of Medicine , Kastamonu University, Kastamonu, Türkiye

**Keywords:** Arteriovenous fistula, Thrombosis, Hemodialysis, Percutaneous thrombolytic therapy, Endovascular intervention

## Abstract

**Background:**

Arteriovenous fistula (AVF) thrombosis remains a critical complication in hemodialysis (HD) patients, often leading to treatment delays and requiring urgent intervention. While endovascular therapy (EVT) is commonly employed, less invasive strategies such as percutaneous thrombolytic therapy are gaining attention due to their potential to restore patency and avoid more complex procedures. This study assessed the effectiveness of percutaneous thrombolytic therapy in acute AVF thrombosis and explored key predictors associated with the need for subsequent endovascular intervention.

**Methods:**

This retrospective study included 42 patients who underwent ultrasound-guided percutaneous thrombolytic therapy using low-dose alteplase (3–5 mg). Technical and clinical success, complication rates, and the need for additional EVT were assessed. Statistical analyses including logistic regression and ROC analysis were used to determine independent predictors for EVT.

**Results:**

The clinical success rate was 97.6%, with 69% of patients achieving AVF patency without EVT. Symptom duration emerged as the strongest predictor for EVT; patients with symptoms > 2.5 days had significantly higher EVT rates (*p* = 0.01). Each additional day of symptoms increased the odds of requiring EVT by 88.5% (OR = 1.885, *p* = 0.012). Female patients were also more likely to require EVT than males (*p* = 0.005). No significant associations were found for age, BMI, or fistula characteristics.

**Conclusion:**

Percutaneous thrombolytic therapy is a highly effective and minimally invasive option for acute AVF thrombosis. Symptom duration > 2.5 days is a key threshold predicting the need for EVT, highlighting the critical importance of early intervention. These findings may inform clinical decision-making and optimize access salvage strategies in dialysis patients.

## Introduction

Vascular access methods in hemodialysis (HD) patients tend to vary over time. Although arteriovenous fistula (AVF) is the first choice for patients starting long-term HD, a significant portion of these patients continue HD via a catheter [1–3]. AVF thrombosis is the most common cause of vascular access problems in HD patients. Thrombus formation can occur immediately after AVF creation, often due to inflow issues, anastomotic stenosis, technical errors, or anatomical abnormalities. In later stages, thrombosis typically arises from outflow venous stenosis or intimal hyperplasia [4]. Urgent intervention is required for patients to continue HD. Treatment options include surgical thrombectomy (open repair) and endovascular therapy (EVT) [5–7].

In recent years, percutaneous thrombolytic therapy has emerged as an alternative or adjunctive treatment, yielding successful outcomes. Studies have demonstrated that this technique is a fast, practical, and repeatable method with a low risk of bleeding, providing a safe treatment option that can help avoid unnecessary EVT or surgical procedures [8–11].

In this study, we aimed to evaluate the efficacy and outcomes of percutaneous thrombolytic therapy in acute AVF thrombosis cases and identify the factors influencing the need for EVT.

## Methods

### Patient selection

In this study, a retrospective analysis was conducted on 42 patients who presented to our clinic between January 2022 and February 2024 and underwent percutaneous thrombolytic therapy for AVF thrombosis. Patients not suitable for treatment were excluded from the study, including those with thrombotic segment lengths longer than 10 cm, those with associated skin infections at the thrombosed segment, and those with contraindications for thrombolytic agents. Informed consent was obtained from all patients. The ethics committee approval was obtained from the Kastamonu University Faculty of Medicine Clinical Researches Ethics Committee (Date: 16/02/2024, Decision No: 2024/38). All procedures performed in this study were conducted in accordance with the ethical standards of the Declaration of Helsinki and relevant institutional guidelines. All patient data were anonymized prior to analysis, and no identifiable personal information was used. There were no missing data for the variables analyzed; all included patients had complete datasets.

### Procedure

The procedures were performed in the interventional radiology suite. The presence of acute thrombus and the length of the thrombosed segment were determined using the Aplio 500 (Toshiba) ultrasound (US) device in our clinic. Percutaneous interventions were performed under ultrasound guidance and sterile conditions. A 27G needle was used to access the thrombosed segment, and 3–5 mg of rtPA (alteplase) was administered as the thrombolytic agent. The dosage was adjusted according to the length of the thrombosed segment. Alteplase was diluted with 0.9% saline at a 1:2 ratio and drawn into 2.5 cc syringes, then slowly injected percutaneously into the thrombosed segment under ultrasound guidance. Multiple percutaneous accesses were made to different regions of the fistula to deliver rtPA, covering the entire thrombus. Afterward, the patient was transferred to the ward for monitoring of bleeding and medical management.

Post-procedure, patients were observed in the ward for 24 h. During this period, patients were administered subcutaneous low molecular weight heparin (LMWH) at renal failure dosage and oral 100 mg acetylsalicylic acid (ASA) [12]. After 24 h, follow-up US was performed, and patients with fully patent AVFs were allowed to continue hemodialysis through the fistula. In cases of residual thrombus, patients were managed with medical therapy (LMWH + ASA) and, if necessary, temporary dialysis catheters were placed to allow hemodialysis. EVT was applied to patients with incomplete patency during follow-up.

Angioplasty for all post-treatment stenoses was performed using 4–8 mm balloon catheters (Mustang, Boston Scientific, MA, USA). Hemostasis was achieved using a modified purse-string suture technique [13]. All interventions were performed by an interventional radiologist with more than 5 years of endovascular experience. The initiation of HD and post-intervention was confirmed through both paper and electronic medical records.

Assessment of AVF patency was performed by an interventional radiologist using Doppler ultrasound. Incomplete patency was defined as persistent residual thrombus or reduced flow deemed insufficient for dialysis access restoration Technical success was defined as palpable thrill post-treatment and complete patency demonstrated by US. Clinical success was defined as restoration of AVF patency sufficient to allow continuation of hemodialysis, confirmed by both a palpable thrill and Doppler ultrasound findings.

### Statistical analysis

Various statistical analyses were conducted to evaluate differences between groups and the effects of independent variables on the need for endovascular procedures. The “Shapiro–Wilk” test was used to assess whether continuous variables followed a normal distribution. For variables that did not follow a normal distribution, the “Mann–Whitney U” test was used, while the “independent samples t-test” was applied for variables with normal distribution. Relationships between categorical variables were analyzed using the “Chi-square” test. Correlations between variables were examined with “Spearman's rho” correlation analysis. Logistic regression analysis was used to identify predictors of the need for EVT. Additionally, “ROC curve analysis” was conducted to determine an optimal threshold value for symptom duration, and patients were grouped according to this threshold. Odds ratios (OR) were calculated, and the statistical significance of differences between groups was evaluated using “cross-tabulation and Chi-square test.” All analyses were performed using SPSS software (IBM SPSS Statistics for Windows, Version 23.0), and results with a *p*-value < 0.05 were considered statistically significant.

## Results

A total of 42 patients underwent percutaneous rtPA therapy in this study, of which 13 required EVT, while 29 did not. One patient who required EVT developed a subcutaneous hematoma that did not necessitate surgical intervention and resolved spontaneously during follow-up. The overall clinical success rate of the procedure was 97.6%, with only one patient requiring a new fistula creation due to incomplete patency.

Among the 42 patients included in the study, the mean age was 61.07 ± 13.19 years, and the mean body mass index (BMI) was 25.67 ± 3.82. The mean fistula duration was 25.52 ± 20.74 months, with a mean thrombosed segment length of 7.14 ± 2.83 cm. Of the patients, 24 (57.14%) were male, and 18 (42.86%) were female. Most patients had left-sided fistulas (30; 71.43%), with distal radiocephalic (21; 50%), proximal radiocephalic (13; 30.95%), and brachiocephalic (8; 19.05%) locations. Comorbidities included diabetes mellitus in 18 patients (42.86%), hypertension in 22 patients (52.38%), and coronary artery disease in 8 patients (19.05%). Regarding medication use, 7 patients (16.67%) were on acetylsalicylic acid (ASA), and 5 patients (11.9%) were on clopidogrel (Tables 1 and 2).

**Table 1:** Comparison of continuous variables between patients requiring and not requiring endovascular intervention

Variable	Total Mean ± SD (min–max)	EIRG Mean ± SD (min–max)	EINRG Mean ± SD (min–max)	*p* value
Count	42	13	29	
Age (y)	61.07 ± 13.19 (23–78)	60.38 ± 16.92 (23–78)	61.37 ± (27–76)	0.66
BMI	25.67 ± 3.82 (19.79–34.75)	26.61 ± 4.23 (21.37–34.48)	25.25 ± 3.62 (19.79–34.75)	0.29
Fistula duration (m)	25.52 ± 20.74 (1–84)	23.00 ± 19.13 (4–71)	26.65 ± 21.65 (1–84)	0.76
Thrombosed segment (cm)	7.14 ± 2.83 (3.2–15.6)	8.23 ± 4.06 (3.2–15.60)	6.65 ± 1.97 (4.3–12.9)	0.36
Symptom duration (d)	3.57 ± 2.22 (1–12)	5.23 ± 2.71 (2–12)	2.82 ± 1.48 (1–6)	0.002

*EIRG* Endovascular intervention required group, *EINRG* Endovascular intervention not-required group, *SD* Standard deviation, *BMI* Body mass index, *y* Years, *m* Months, *d* Days

**Table 2:** Comparison of categorical variables between patients requiring and not requiring endovascular intervention

Variable	Category	Total (*n* = 42)	EIRG (*n* = 13)	EINRG (*n* = 29)	*p* value
Sex	Female	18 (42.86%)	10 (76.92%)	8 (27.59%)	0.005
	Male	24 (57.14%)	3 (23.08%)	21 (72.41%)	
Side	Left	30 (71.43)	11 (84.62%)	19 (65.52%)	0.28
	Right	12 (28.57%)	2 (15.38%)	10 (34.48%)	
Location	BC	8 (19.05%)	2 (15.38%)	6 (20.69%)	0.60
	Proximal RC	13 (30.95%)	3 (23.08%)	10 (34.48%)	
	Distal RC	21 (50%)	8 (61.54%)	13 (44.83%)	
Comorbidity	DM	18 (42.86%)	5 (38.46%)	13 (44.83%)	0.70
	HT	22 (52.38%)	8 (61.54%)	14 (48.28%)	0.42
	CAD	8 (19.05%)	2 (15.38%)	6 (20.69%)	0.68
Drug	ASA	7 (16.67%)	2 (15.38%)	5 (17.24%)	0.88
	Klopidogrel	5 (11.90%)	2 (15.38%)	3 (10.34%)	0.64
Fistul age	< 1 year	13 (31%)	4 (30.77%)	9 (31.03%)	0.98
	> 1 year	29 (69%)	9 (69.23%)	20 (68.97%)	
Symptom duration	< 2.5 days	15 (35.71%)	1 (7.69%)	14 (48.28%)	0.01
	> 2.5 days	27 (64.29%)	12 (92.31%)	15 (51.72%)	

*EIRG* Endovascular intervention required group, *EINRG* Endovascular intervention not-required group, *BC* Brachiocephalic, *RC* Radiocephalic, *CAD* Coronary artery disease, *HT* Hypertension, *DM* Diabetes mellitus, *ASA* Asetil salisilik asit

The comparison of continuous variables between patients requiring and not requiring EVT revealed that the mean symptom duration was significantly longer in the EVT-requiring group (5.23 ± 2.71 days) compared to the non-requiring group (2.82 ± 1.48 days; *p* = 0.002). However, there were no significant differences in the mean age, fistula duration or thrombosed segment length between the groups (*p* > 0.05) (Table 1).

**Fig. 1:**
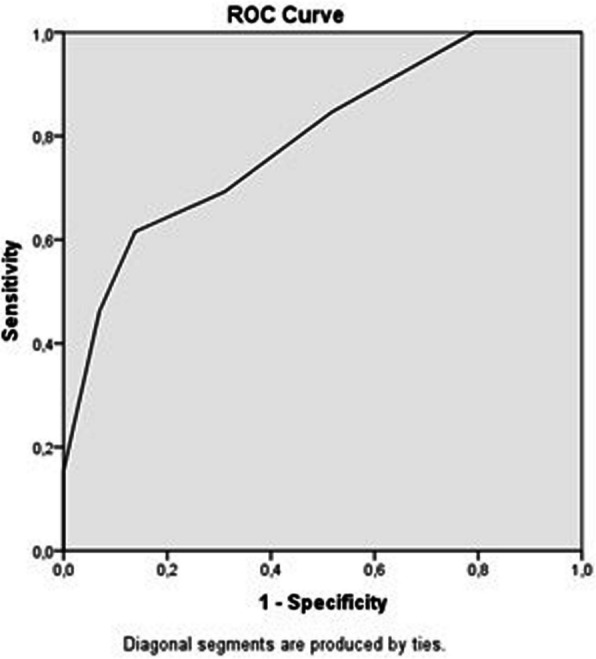
Evaluation of the predictive power of symptom duration on the requirement for endovascular procedures using ROC analysis. Receiver operating characteristic (ROC) curve demonstrating the diagnostic ability of symptom duration (in days) to predict the need for an endovascular procedure. The area under the curve (AUC) is 0.792 (95% CI: 0.641–0.942; *p* = 0.003), indicating a good predictive power of the symptom duration

ROC curve analysis determined an optimal threshold value of 2.5 days for symptom duration, with an area under the curve (AUC) of 0.792 (95% CI: 0.641–0.942; *p* = 0.003), indicating good predictive power (Fig. 1). When evaluating the need for EVT according to categorical variables such as gender, fistula side, fistula location, comorbidities and symptom duration (with a threshold of 2.5 days), it was found that female patients required EVT more frequently than males (*p* = 0.005). Additionally, patients with a symptom duration of greater than 2.5 days also required EVT more frequently (*p* = 0.01). No significant differences were found for the other parameters. (Table 2).

Spearman correlation analysis was conducted to assess the presence of correlations between symptom duration and other clinical parameters. The analysis revealed that symptom duration did not demonstrate a statistically significant correlation with the other parameters (*p* > 0.05) (Table 3).

**Table 3:** Spearman correlation between symptom duration and other clinical parameters

	Correlation coefficient	*p* value
Age	0.07	0.65
BMI	0.15	0.32
Fistula duration	0.24	0.11
Thrombosed segment	0.27	0.08

*BMI* Body mass index

The logistic regression analysis assessed the factors influencing the requirement for EVT. The effects of age (*p* = 0.534), fistula duration (*p* = 0.262) and the thrombozed segment length (*p* = 0.357) on the need for EVT were found to be statistically insignificant. However, a significant positive association was identified between symptom duration and the necessity for EVT (*p* = 0.012); specifically, each additional day of symptom duration increased the odds of requiring an EVT by 88.5% (Table 4).

**Table 4:** Logistic regression analysis of factors influencing the requirement for endovascular intervention

Variable	SD	OR	95% CI	*p* value
Age	0.038	1.024	0.951–1.103	0.534
Fistula duration (m)	0.026	0.970	0.922–1.021	0.262
Thrombozed segment (cm)	0.197	1.198	0.815–1.763	0.357
Symptom duration (d)	0.251	1.885	1.153–3.083	0.012

*SD* Standard deviation, *OR* Odds ratio, *CI* Confidence interval, *m* Months, *d* Days

## Discussion

In this study, it was determined that exceeding a symptom duration of 2.5 days was a critical factor increasing the need for EVT, highlighting symptom duration as an important predictor of the requirement for such procedures. This finding provides a crucial criterion that can guide clinical decision-making, particularly in cases where early intervention is necessary.

Thrombolytic therapy in AVFs is generally administered via catheter, and challenges such as bleeding complications and difficulty in achieving hemostasis can occur with this method. The strong fibrinolytic effect of thrombolytic agents increases the risk of bleeding from previous access sites and prolongs the hemostasis process. Delayed bleeding and hematomas related to the use of tPA have been reported in the literature [14, 15]. Regus et al. demonstrated that prolonged tPA exposure reduced complication rates and facilitated EVT [16]. Based on this information, we used lower doses of tPA (3–5 mg) in our clinic and monitored patients in the ward until the next day after confirming minimal blood flow with Doppler US. In most patients, sufficient blood flow for HD was achieved as confirmed by follow-up US. Additionally, we successfully achieved hemostasis using the suture technique in patients who underwent EVT post-thrombolytic therapy. As a result of this approach, our treatment success rate was high (97.6%) and the complication rate low (2.4%) compared to previous studies.

The underlying causes of AVF thrombosis typically include fistula stenosis and aneurysms, and previous studies have found the efficacy of thrombolytic therapy alone to be limited [17]. Schon et al. emphasized that while successful outcomes were achieved using low doses of tPA, the most important factor for treatment success was EVT, particularly balloon angioplasty [18, 19]. Additionally, studies have reported similar outcomes between PTA and pharmacomechanical thromboaspiration [20, 21]. Our patient group consisted of those who experienced acute fistula failure due to thrombosis and had no prior issues during HD sessions. In our percutaneous thrombolytic treatment approach, fistula patency was restored in 29 of 42 patients without the need for EVT. We attributed this to the fact that the patients had no previous issues during HD sessions and that the fistula returned to normal function after the thrombus was removed.

Thrombosis progresses in four stages: induction, acute fibrin-dominant, intermediate, and chronic connective tissue-dominant phases. The duration and structure of thrombosis affect its responsiveness to treatment. Thrombi are more responsive to thrombolytic therapy in the early fibrin-dominant stages, while they become more resistant with the organization process in later stages [22]. While symptom duration is frequently used to estimate thrombus age, recent evidence suggests that it may not always correlate with the degree of thrombus organization. A subanalysis of the ClotTriever Outcomes registry demonstrated that in 55% of cases, the actual thrombus morphology was more chronic than suggested by the patient-reported duration of symptoms. This discrepancy implies that organizational changes within the thrombus may begin earlier or progress more rapidly than clinically assumed, thereby reducing responsiveness to thrombolytic therapy even in patients with seemingly “short” symptom durations [23]. These findings reinforce the notion that early intervention remains critical, not only due to elapsed time but also due to the biological variability in thrombus evolution. Although clot echogenicity was not systematically classified in this study, it may serve as a noninvasive surrogate marker of thrombus organization and should be explored in future research.

In our study, it was shown that symptom duration exceeding 2.5 days increased the need for EVT. This finding underscores the importance of early diagnosis and treatment decisions, suggesting that organizational changes in the thrombosed segment may increase the difficulty of intervention. Umanath et al. demonstrated the advantages of early thrombolytic therapy by reporting a 73.8% success rate and a 5.6% complication rate in 321 procedures performed by nurses on 145 patients, highlighting the safety and efficacy of this approach [24]. These findings suggest that early management of thrombosis, particularly interventions before clot organization begins, can enhance treatment success.

High BMI and obesity have been associated with worsened AVF maturation outcomes, lower primary patency rates, and higher reintervention needs [25]. There is no significant relationship between advanced age and AVF primary patency, maturation, complications, or intervention needs [26]. In our study, no statistically significant relationship was found between BMI, age, and the need for EVT.

Our study observed that female patients required endovascular intervention more frequently than males, a finding also supported in the literature. Lee et al. highlighted that microvascular dysfunction is more common in women than in men, increasing the need for EVT [27]. Similarly, Beale et al. showed that hormonal changes and increased inflammatory responses raise the risk of vascular complications in women [28]. While these factors may help contextualize our findings, further studies are needed to clarify the underlying mechanisms behind the increased EVT requirement observed in female patients.

Rodrigues et al. reported that fistulas with a duration of less than one year more frequently required reintervention [29]. Similarly, while no significant relationship was found between fistula duration and the need for EVT in our study, it was observed that fistulas with shorter durations carried a higher risk for intervention. Our correlation analysis also found a significant positive correlation between fistula duration and thrombotic segment length. Additionally, the positive correlation between alteplase dose and thrombotic segment length may be an important finding that could influence treatment strategies. Further studies with larger patient populations are needed to emphasize the clinical significance of this relationship.

### Limitations

This study has several limitations, including its retrospective design, single-center setting, and relatively small sample size. The absence of a control group, such as patients treated with EVT-only or alternative thrombolytic dosing strategies, limits comparative interpretation of outcomes. Moreover, data regarding inflammatory or coagulation markers, as well as prior AVF interventions, were not consistently available in medical records and therefore could not be included in the analysis. However, it is noteworthy that this study includes one of the largest patient populations on this topic. The use of ROC analysis to determine an optimal threshold for symptom duration is one of the study's strengths, and it may contribute significantly to clinical decision-making processes.

## Conclusion

The results of this study demonstrate that percutaneous thrombolytic therapy is an effective and safe method for managing AVF thrombosis and that early intervention can reduce the need for EVT. Symptom duration exceeding 2.5 days is an important factor that increases the need for EVT. Prospective studies with larger patient groups are needed to confirm the findings and optimize treatment strategies.

## Data Availability

The datasets generated and/or analyzed during the current study are not publicly available due to ethical reasons but are available from the corresponding author on reasonable request.
